# High-Resolution Detection of Microplastics in Zooplankton from Lake Como (Northern Italy): A Multi-Year Baseline for Large Deep Lakes

**DOI:** 10.3390/toxics14040342

**Published:** 2026-04-19

**Authors:** Benedetta Villa, Gaia Bolla, Ginevra Boldrocchi, Roberta Bettinetti

**Affiliations:** 1 Department of Science and High Technology, University of Insubria, Via Valleggio 11, 22100 Como, Italy; bvilla3@uninsubria.it (B.V.);; 2 Department of Human Sciences, Innovation and Territory, University of Insubria, Via Valleggio 11, 22100 Como, Italy; ginevra.boldrocchi@uninsubria.it

**Keywords:** microplastics (MPs), zooplankton, Lake Como, freshwater ecosystems, subalpine lakes, temporal and seasonal variability, Nile Red and Rose Bengal staining, high-resolution microscopy

## Abstract

Microplastics (MPs) are emerging contaminants in freshwater ecosystems, yet their ingestion by zooplankton remains poorly documented in large European lakes. This study provides the first evidence of MPs in zooplankton from Lake Como (Northern Italy), a major subalpine lake of ecological and socioeconomic relevance. Using high-resolution digital microscopy (detection limit: 2 µm), we quantified MPs across four sampling years (2016, 2017, 2018, 2025), capturing small size fractions typically overlooked by conventional methods. MPs were consistently detected, with mean concentrations of 0.06 ± 0.08 MPs ind.^−1^ and 1.14 ± 1.22 MPs mg^−1^ d.w., values comparable to those reported for freshwater zooplankton worldwide. No significant differences were observed between the lake’s two main branches, supporting a lake-wide interpretation of exposure. Clear seasonal patterns emerged, with higher MPs loads in autumn and winter. These findings highlight the potential for MPs to enter pelagic food webs and contribute to a lake-wide baseline for future harmonized monitoring and polymer-specific assessments. The main limitation of this study is the exclusive quantitative approach, which does not provide qualitative information on polymer composition. Overall, these results underscore the need to integrate zooplankton-based monitoring into freshwater microplastic risk assessment frameworks.

## 1. Introduction

Microplastics (MPs) are now pervasive contaminants across terrestrial, freshwater and marine ecosystems, posing multifaceted ecological risks. Their marked physical and chemical heterogeneity (ranging in size from 5 mm down to 0.1 µm) [1,2], together with a broad variety of polymers, additives, and morphologies, strongly influences their toxicological properties, environmental transport, and fate [3]. Recent studies have highlighted how environmental aging, UV degradation and biofilm colonization further modify MPs’ surface chemistry, increasing their capacity to absorb metals, PFAS and organic pollutants [4,5]. These characteristics complicate accurate analysis, identification and quantification, underscoring the need for coordinated efforts to map contamination patterns across ecosystems.

Since their first detection in lakes in 2013 [6], a growing body of evidence has shown that MPs can affect freshwater ecosystems and their biota [7,8,9,10,11,12]. However, compared to marine environments, where systematic research began in the 1970s and accounts for about 62% of studies, freshwater monitoring remains relatively limited [13,14,15,16]. This gap has been repeatedly emphasized in recent reviews, which called for harmonized freshwater monitoring frameworks and standardized analytical protocols [15,17].

Lakes and rivers are often tightly connected to point sources such as wastewater treatment plants, dense urban centers and industrial shorelines [18], and because lakes have smaller volumes than seas, they can concentrate MPs more locally [19,20]. Recent global assessments confirm that lakes can reach MP concentrations comparable to or exceeding those of coastal marine environments, especially in regions with high tourism pressure, textile industries or intensive agriculture [17].

Laboratory studies have documented a wide range of sublethal effects of MPs on fishes and other freshwater organisms: altered gene expression linked to oxidative stress, inflammation, stress responses and reproduction [21,22,23,24] and other types of damage to various organs [25,26,27,28]. Reported individual-level responses include shifts in body condition and relative organ sizes [27,29,30]. However, many of these studies relied on single-species laboratory exposures that use single polymer types and particle sizes, often at concentrations well above those typically measured in the environment [31,32]. Recent ecotoxicological research has stressed the need for more realistic exposure scenarios, incorporating environmentally aged particles, polymer mixtures, and complex contaminant cocktails that better reflect real-world conditions [33].

Lake Como, one of the main Italian subalpine lakes, lies in a heavily industrialized and densely populated region, and therefore represents a complex real-world exposure setting. The lake has high regional ecological and socioeconomic value: it provides drinking water for surrounding municipalities, supports tourism that attracts millions of visitors annually, sustains local fisheries and agricultural activities, and helps regulate water resources for downstream industrial and irrigation uses across the Adda River basin [34,35,36]. Given these roles, understanding MP dynamics in Lake Como is crucial for local stakeholders and contributes to global assessments of inland lake contamination.

Potential sources of MPs to Lake Como mirror the diversity of human activities around the lake (wastewater treatment plants, urban runoff and landfills, industrial discharges, fisheries and agricultural inputs, and atmospheric transport). Consequently, contamination patterns reflect both urban and rural influences [15,37,38,39]. Despite its importance, MP contamination in Lake Como remains understudied: only a few studies have assessed MPs in sediments and water [40,41,42,43], and to date, only one study has examined MPs in fish species (*Perca fluviatilis*) [11]. Zooplankton, despite being key bioindicators and active vectors for transferring MPs into food webs, have never been surveyed for MPs in Lake Como, even though numerous studies demonstrate their ingestion of MPs and their role in trophic transfer [44,45,46,47,48,49,50]. Recent studies have reinforced the central role of zooplankton in the vertical and horizontal redistribution of MPs in lakes [7,10,51].

This study therefore fills a critical knowledge gap by quantifying, for the first time, the presence and concentrations of MPs in zooplankton from Lake Como. This baseline dataset provides essential information to assess potential implications for ecosystem health and food-web dynamics and will improve comparability with other lacustrine systems worldwide. By situating Lake Como within the broader global context of inland-water contamination, the work contributes regionally relevant evidence that can inform monitoring priorities, risk assessment and management of MPs in freshwater resources.

## 2. Materials and Method

### 2.1. Study Area

**Figure 1:**
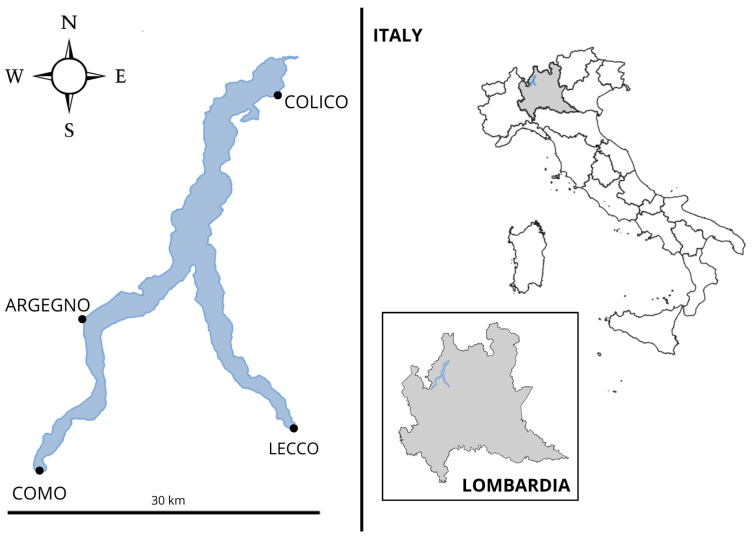
Map of Lake Como and principal cities (figure made by the authors).

Lake Como, located in northern Lombardy (Italy) between the provinces of Como and Lecco, lies at approximately 46°10′ N and 09°16′ E. It represents the deepest hydrographic basin in Italy, with a maximum depth of 425 m at Argegno. The lake covers an area of about 4500 km^2^, with a total volume of 22.5 km^3^ and a surface area of 145 km^2^. Its morphology is characterized by an inverted Y-shape consisting of three main sub-basins—northern, western, and eastern (Figure 1). The lake has a single outlet, the Adda River, located near Lecco in the eastern sub-basin, resulting in a relatively long water renewal time of about 12.8 years [39,52]. Water renewal times differ substantially between the western and eastern basins due to their distinct morphological and hydrodynamic features [53]. Lake Como is classified as oligomictic, with complete water column mixing occurring only during years with cold and windy winters.

### 2.2. Zooplankton Sampling

Zooplanktonic organisms were seasonally sampled over three consecutive years (2016–2018) by Pascariello et al. [36] and again in 2025 (Table S2). The 2016–2018 data establish a multi-year baseline, while the 2025 samples provide a contemporary snapshot. Archived aliquots from 2016 to 2018 were re-processed in 2025 using consistent analytical protocols to enable direct comparability, despite the seven-year gap with no intermediate sampling due to resource constraints. Zooplankton were vertically collected from the surface to a depth of 20 m using a 200-µm mesh nylon zooplankton net (52 cm mouth diameter), targeting dominant mesozooplankton taxa and ensuring sufficient biomass for MPs quantification.

### 2.3. Microplastics Analysis

Once in the laboratory, density and biomass were determined under an optical microscope based on length–weight regression equations derived from Lake Como samples. Then, organisms were rinsed on a stainless-steel 200 µm sieve with MilliQ water to minimize residual MPs adhering to the organisms.

For MPs analysis, aliquots were collected from a beaker under magnetic stirring at mid-depth, halfway between the vortex center and the vessel wall, following common sampling practices [54] to ensure representativeness. The aliquots were dried in an oven at 40 °C for 48 h to determine dry weight (d.w.), with an average of 0.04 ± 0.09 g d.w. of zooplankton used per aliquot. Samples were then subjected to oxidative digestion with 25 mL of 30% analytical-grade hydrogen peroxide (Carlo Erba, Milan, Italy) for 48 h at 40 °C to remove organic matter without affecting synthetic polymers [55,56]. The digested material was vacuum-filtered through a Büchner funnel using glass fiber filters (Whatman^®^ glass microfiber grade GF/C filter discs, 1.2 μm pore size, 47 mm diameter, Cytiva, Marlborough, MA, USA).

MP quantification was carried out using an optical digital microscope (Keyence VHX-7000, Osaka, Japan) with a resolution of up to 2 µm, allowing the detection of particles from 2 µm to 5 mm, and combined with staining techniques, as described in Villa et al. [57]. Briefly, filtered samples were stained with 1–2 mL of Nile Red (99% pure, Carlo Erba, Milan, Italy) dissolved in acetone (99.95% pure, Carlo Erba, Milan, Italy) at 1 g L^−1^, then incubated at 40 °C for 30 min. Filters were then rinsed with 10 mL of acetone followed by 10 mL of MilliQ to remove excess dye, restained with 1–2 mL of Rose Bengal (90% pure, Carlo Erba, Milan, Italy) in MilliQ water (200 mg L^−1^) for 5 min at room temperature and washed again with MilliQ water. Subsequently, the stained filters were examined under the digital microscope to quantify the presence of MPs.

Following Villa et al. [57], the Nile Red/Rose Bengal co-staining method enables differentiation of MPs from natural polymers and allows consistent visual detection and quantification of particles down to 2 μm. However, this approach does not permit polymer identification; therefore, all MPs counts should be considered presumptive identifications pending future spectroscopic confirmation (FTIR/Raman). In addition, the staining performance on environmentally aged MPs or particles with biofilm development remains uncertain, as these alterations may modify dye interactions compared to pristine polymers [58].

Standard MP anti-contamination measures were applied, including the use of cotton lab coats, working under a laminar flow hood whenever possible, and minimizing plastic equipment. Work surfaces and glassware were thoroughly cleaned before use, all solutions were prefiltered, and sample containers were kept covered to reduce airborne particle contamination. Procedural blanks (one per 6–7 samples per analytical batch) were processed alongside zooplankton samples following the identical protocol—including all filtration, digestion, density separation, and microscopy steps—to detect and correct for potential contamination. The total particles (fibers + fragments) detected in each blank (Table S4) were subtracted from the corresponding sample counts within the same analytical batch. LOD and LOQ were calculated as mean + 3 × SD and mean + 10 × SD of procedural blank counts, respectively, yielding LOD = 8.99 and LOQ = 24.53 MP sample^−1^. Five samples below LOD (Table S1) were treated as zero for statistical analyses adopting a conservative approach prioritizing confirmed detections over potential overestimation. Blanks subtraction introduced an uncertainty of 2.22.

Results are reported as MPs per individual (MPs ind.^−1^) and MPs per mg (MPs mg^−1^) d.w.

### 2.4. Statistical Analysis

Differences in MP concentrations (MPs ind.^−1^ and MPs mg^−1^) between lake branches (Como vs. Lecco) were tested using independent-sample *t*-tests. Differences among multiple groups (sampling years: 2016, 2017, 2018, 2025; seasons: spring, summer, autumn, winter) were evaluated using one-way ANOVA followed by post hoc Tukey’s HSD tests when normality and homogeneity of variances assumptions were met (verified via Shapiro–Wilk and Levene’s tests, respectively). Non-parametric Kruskal–Wallis tests were applied when assumptions were violated. Statistical significance was set at *p* < 0.05. All analyses were performed using XLSTAT v.2025.1.3 (Addinsoft, New York, NY, USA) in Microsoft Excel.

## 3. Results

All results are reported in Table S1 of the Supplementary Information, while zooplankton density data are provided in Supplementary Text S2.

Lake Como showed overall means of 0.06 ± 0.08 MPs ind.^−1^ and 1.14 ± 1.22 MPs mg^−1^ d.w. across all samples, with particles sizes ranging from 2 to 1680 μm [12].

The high-resolution digital microscopy employed in this study enabled the detection of MPs from 5 mm down to 2 µm, substantially reducing underestimation bias typically associated with conventional methods and capturing the ecologically relevant 20–2 µm fraction often missed due to instrumental limitations [3,55,56].

**Figure 2:**
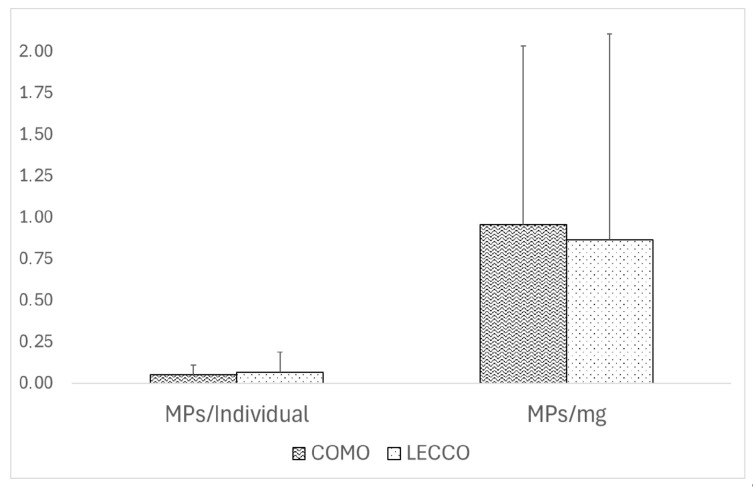
Mean (±SD) of MP levels (MPs ind.^−1^ and MPs mg^−1^ d.w.) in the Como and Lecco branches of Lake Como (2016, 2017, 2018).

Mean MP contamination levels were 0.05 ± 0.06 MPs ind.^−1^ in the Como branch (n = 19) and 0.07 ± 0.12 MPs ind.^−1^ in the Lecco branch, with Lecco showing the highest recorded value of 0.37 MPs ind.^−1^ (Figure 2). For mass-normalized concentrations, the Como branch averaged 0.96 ± 1.08 MPs mg^−1^, while Lecco exhibited 0.86 ± 1.24 MPs mg^−1^ with a maximum of 4.00 MPs mg^−1^ (Figure 2).

The Como sub-basin exhibited a fragment proportion of 73.3% and fibers of 26.7%, while Lecco sub-basin showed a similar predominance of fragments (71.3%) over fibers (28.7%) in zooplankton samples.

Only five samples were below the LOD (three at Lecco and two at Como), and their balanced distribution between branches suggests a negligible influence on spatial comparison.

Despite the morphological and hydrodynamic differences between the two basins, Lake Como was treated as a single system in this study, as no significant spatial differences were detected for either MPs ind.^−1^ or MPs mg^−1^ (*p* > 0.05; Figure 2). This approach enabled robust temporal and seasonal analyses and facilitated comparisons with other freshwater ecosystems.

**Figure 3:**
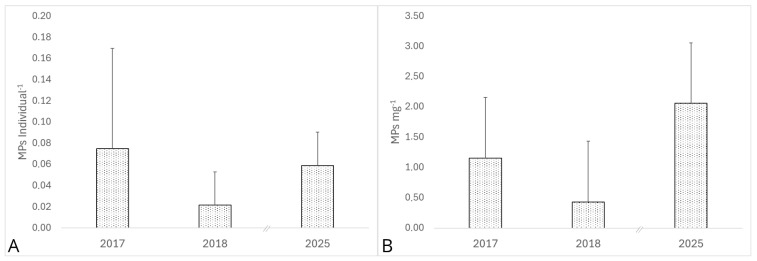
Mean (± SD) of MPs levels: (**A**) MPs ind.^−1^ and (**B**) MPs mg^−1^ d.w. in Lake Como among years 2017, 2018 and 2025 (// indicates 7-year sampling gap). Data from 2016 were excluded due to substantially lower sample sizes compared to 2017–2018, providing insufficient statistical power for temporal variability analysis.

Mean MP ind.^−1^ concentrations were 0.07 ± 0.09 (2017), 0.02 ± 0.03 (2018), and 0.06 ± 0.03 (2025). For MPs mg^−1^, values stood at 1.15 ± 1.25 (2017), 0.43 ± 0.57 (2018), and 2.06 ± 1.10 (2025) (Figure 3).

Across all years, fragments consistently dominated MP morphology, while fibers accounted for the remaining fraction. In 2016, fragments and fibers accounted for 65% and 35% of items, respectively. In 2017, the proportion of fragments increased to 74.5%, with fibers decreasing to 25.5%. In 2018, fragments comprised 72.8% of the particles, compared to 27.2% for fibers. In 2025, fragments still prevailed at 71.9%, whereas fibers represented 28.1% of the total.

Temporal analyses revealed no significant interannual differences in MPs ind.^−1^ (*p* > 0.05), whereas MPs mg^−1^ exhibited marked variability, with a decrease in 2018 followed by elevated levels in 2025 (*p* < 0.05; Figure 3). These fluctuations occurred independently of major changes in zooplankton density (Supplementary Text S2), suggesting that biomass normalized metrics are more sensitive to temporal exposure dynamics. Similar interannual variability has been reported in other freshwater systems, attributed to hydrological conditions, runoff intensity, and seasonal tourism [59,60].

**Figure 4:**
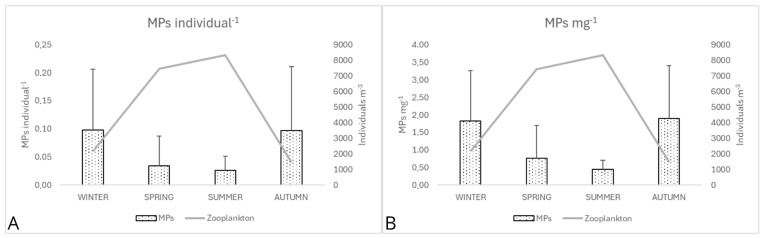
Seasonal levels of MPs in zooplankton, expressed as (**A**) MPs ind.^−1^ and (**B**) MPs mg^−1^ d.w. across all years 2016, 2017, 2018 and 2025.

MPs ind.^−1^ varied seasonally from 0.098 ± 0.109 (winter) and 0.097 ± 0.114 (autumn) to lower values in spring (0.034 ± 0.053) and summer (0.026 ± 0.025), while MPs mg^−1^ peaked in autumn (1.902 ± 1.502) and winter (1.834 ± 1.420), declining through spring (0.764 ± 0.939) to summer (0.457 ± 0.253) (Figure 4).

Fragments consistently dominated MP morphology across all seasons, comprising most particles, while fibers formed the minority fraction. Specifically, winter showed 65.5% fragments and 34.5% fibers; spring exhibited 74.7% fragments and 25.3% fibers; summer had 71.7% fragments versus 28.3% fibers; and autumn peaked at 75.2% fragments and 24.8% fibers. Seasonal patterns were statistically significant: MPs mg^−1^ were higher in autumn and winter, while summer exhibited significantly lower values (*p* < 0.05; Figure 4). These trends align with established seasonal dynamics of MP distribution and zooplankton ecology in temperate lakes [36,52].

## 4. Discussion

This study provides the first evidence of MP ingestion by zooplankton in Lake Como, confirming their role as effective sentinels of MPs exposure and their capacity to mediate trophic transfer within pelagic food webs [44,45,46,47,48,49,50,61].

High-resolution digital microscopy enabled detection of particles as small as 2 µm, capturing the frequently overlooked 20–2 µm size range and reducing underestimation biases typical of standard methods [3,55,56]. This analytical advantage strengthens the reliability of the baseline established for Lake Como and supports interpretation of MPs ingestion across seasons and years. Nonetheless, as this approach does not allow polymer identification, all MPs counts should be regarded as preliminary and subject to spectroscopic confirmation (FTIR/Raman) in future analyses.

The absence of significant spatial differences in MP concentrations (both MPs ind.^−1^ and MPs mg^−1^) between the morphologically distinct Como and Lecco branches [41,53] is consistent with the possibility that lake-wide mixing processes dominate over local hydrodynamic barriers [62]. However, given that the statistical comparison is based on a relatively small number of samples (*n* = 19 Como; *n* = 18 Lecco; 5 < LOD: 3 at Lecco, 2 at Como), this pattern should be interpreted cautiously. Hypothesized contributing factors might include inefficient wastewater treatment [35,39,63], possible atmospheric deposition [64], potential sediment resuspension [65], and probable stormwater runoff [41]. However, none of these processes were directly measured in this study. These interpretations should therefore be considered speculative working hypotheses, supported by patterns observed in other large lakes under mixed urban–rural pressures [7,17].

Temporal variability revealed relatively stable MPs ind.^−1^ levels across years (means: 0.07 ± 0.09 in 2017, 0.02 ± 0.03 in 2018, 0.06 ± 0.03 in 2025), whereas MPs mg^−1^ exhibited pronounced interannual fluctuations, with lower values in 2018 and elevated levels 2025. However, the seven-year gap between 2018 and 2025 precludes interpretation of this as a directional trend; the apparent increase may potentially be influenced by episodic drivers including intensified runoff or tourism peaks, as hypothesized in temperate lakes where hydrological regimes and land-use dynamics have been shown to affect annual MP budgets [59,60]. The absence of consistent temporal trends highlights the dynamic nature of MPs in large lakes and emphasizes the need for long-term monitoring to capture multi-year variability.

Seasonal dynamics were more pronounced, with MP peaks in colder months (autumn/winter) and declining in warmer periods (spring/summer). Plausible explanations for these patterns include lower zooplankton biomass during cold seasons potentially increasing MPs per unit, and winter mixing events possibly resuspending particles from deeper layers, to enhance encounter rates. Conversely, higher spring–summer biomass might dilute MPs across larger populations [36,52]. These patterns are consistent with seasonal cycles of MPs distribution and zooplankton ecology reported in other temperate lakes [61,66,67], although resuspension was not directly measured in this study. All proposed mechanisms remain hypothesis-generating, and require direct correlative data for causal validation.

When placed in a global context (Table 1), MPs levels in Lake Como fall within ranges reported for other lakes and estuarine systems worldwide, including Canadian lakes [68], Lake Kolavai in India [69], Mexican lakes Cuitzeo and Pátzcuaro [70], and estuarine systems in Malaysia and India [71,72]. However, the global dataset on MP pollution in freshwater zooplankton remains geographically biased toward temperate regions in Europe and North America, with sparse coverage of tropical, subtropical, and remote ecoregions. This limitation is compounded by methodological heterogeneity, such as variations in sampling, particle size cutoffs, digestion protocols and analytical techniques, that hinders direct study comparability. These challenges underscore the urgent need for harmonized monitoring frameworks, including standardized protocols for sample collection, processing and analysis, to enable robust global risk assessments and inform effective mitigation strategies [13,15,16]. Within this context, Lake Como provides a valuable baseline for large, deep, subalpine lakes, which are globally relevant yet underrepresented in MP research.

To the best of our knowledge, data on MP concentrations in Lake Como zooplankton—particularly biomass-normalized MPs mg^−1^—remain scarce or entirely absent from the published literature. Existing studies predominantly report MPs per individual, with only one study reporting values from 0.001 to 0.062 MPs L^−1^ [73], reflecting bulk contamination rather than organism-specific ingestion.

Nevertheless, the available literature remains too limited to establish reliable global trends in MP bioaccumulation or accurately benchmark Lake Como against broader patterns.

The multi-year dataset established here (across seasons, years, and lake branches) provides essential data to inform harmonized monitoring frameworks and freshwater MP risk-assessment strategies. Future research should integrate polymer-specific identification (FTIR, Raman), ecotoxicological assessments under environmentally realistic conditions, and expanded spatio-temporal sampling. These efforts will enhance understanding of MP dynamics in deep subalpine basins and support evidence-based management of freshwater resources.

## 5. Conclusions

This study offers the first evidence of MP contamination by zooplankton in Lake Como zooplankton, establishing quantitative baseline concentrations (MPs ind^−1^ and MPs mg^−1^ d.w.) for future monitoring efforts. Our findings confirm that zooplankton readily ingest MPs, underscoring their role as effective sentinels of MP exposure and as key vectors for trophic transfer. The high-resolution analytical approach enabled detection down to 2 µm, capturing the important 20–2 µm size fraction frequently missed by conventional methods.

Mean MP concentrations in zooplankton were comparable to global freshwater systems, highlighting Lake Como’s relevance as an internationally significant freshwater resource. These findings are particularly pertinent for large, deep lakes—freshwater ecosystems markedly underrepresented in MP research.

By providing zooplankton baselines available for such environments, this study contributes essential evidence for understanding MP dynamics in deep, stratified basins and supports the development of monitoring frameworks tailored to these globally important yet understudied systems. These baselines can support regional water authorities in designing long-term monitoring strategies for Lake Como.

Building this foundation through long-term monitoring, ecotoxicological investigations and polymer confirmation via FTIR or Raman spectroscopy will further strengthen future assessments.

## Figures and Tables

**Table 1:** Summary of the studies analyzing MP levels in zooplankton from worldwide freshwater ecosystems.

Location	MPs ind.^−1^	MPs mg^−1^	MPs m^−3^	Reference
Lake Como, Italy	0.06 ± 0.08 (0–0.37)	1.14 ± 1.22 (0–4)	119.5 ± 135.4 (0–541.5)	This study
Lake Mjøsa, Norway	(0.001–0.062 MPs L^−1^)			[73]
Eight lakes in British Columbia, Canada (Alta, Brohm, Chilliwack, Cowichan, Cultus, Deer, Lizard, Pixie)	0.01 ± 0.011 (MPs/copepod) 0.02 ± 0.014 (MPs/Daphnia)			[68]
Lake Kolavai, India	0.17 ± 0.07			[69]
Lake Cuitzeo, Mexico	0.12 ± 0.023			[70]
Lake Pátzcuaro, Mexico	0.105 ± 0.033			[70]
Kuala Terengganu estuarine system, Malaysia	0.022 (0.015–0.037)		9.375 (4.1–20.8)	[72]
Kochi Backwaters estuarine system, India *	0.041 ± 0.035 (0–0.13)			[71]

* strongly fluctuating salinity conditions.

## Data Availability

All data are present in this paper or in the Supplementary Information.

## Supplementary Materials

The supporting information can be downloaded at https://www.mdpi.com/article/10.3390/toxics14040342/s1, Table S1. Overview of zooplankton samples collected in Lake Como between 2016 and 2025. For each sampling event, the table reports the date, branch, sample dry weight (g), number of individuals per aliquot analyzed, estimated zooplankton density (individuals m^−3^), nylon net mesh size (µm), total MP count and their categorization into fragments and fibers. The table also includes normalized values of MPs ind.^−1^ and MPs mg^−1^ d.w. Table S2. Information on zooplankton samples collected in 2025. Table S3. Seasonal variation in MPs contamination of zooplankton (MPs ind.^−1^ and MPs mg^−1^ dry weight) (years 2016, 2017, 2018 and 2025). Table S4. Results of procedural blanks. Text S1. Equation for zooplankton observed exposure to MPs (MPs m^−3^). Text S2. Information on zooplankton density (individual m^−3^) in Lake Como.
