# Supplementary material for: High-Resolution Detection of Microplastics in Zooplankton from Lake Como (Northern Italy): A Multi-Year Baseline for Large Deep Lakes
Source: Toxics. 2026 Apr 19;14(4):342. doi: 10.3390/toxics14040342 (PMC13120432; doi:10.3390/toxics14040342)
Supplement: Supplementary file 1 [file toxics-14-00342-s001.zip › toxics-4217846-supplementary.pdf]

Supplementary information for

Detection of microplastics in zooplankton from Lake Como, Northern Italy: a first record

Benedetta Villa[a], Gaia Bolla[a], Ginevra Boldrocchi[b] and Roberta Bettinetti[b]*

[a]Department of Science and High Technology, University of Insubria, Via Valleggio 11, Como, Italy

[b]Department of Human Sciences, Innovation and Territory, University of Insubria, Via Valleggio, 11, Como, Italy

*Corresponding author: roberta.bettinetti@uninsubria.it

Table of Contents

Table S1 2
Table S2 3
Table S3 3
Table S4 3
Text S1 4
Text S2 4
References 4

Table S1. Overview of zooplankton samples collected in Lake Como between 2016 and 2025. For each sampling event, the table reports the date, branch, sample dry weight (g), number of individuals per aliquot analysed, estimated zooplankton density (individuals m^{-3}), nylon net mesh size (µm), total MP count and their categorization into fragments and fibers. The table also includes normalized values of MPs $ind.^{-1}$ and MPs mg^{-1} d.w.

Date	Como Lake Branch	Dry Weight (g)	Individuals per aliquot	Individuals m^{-3}	Mesh size (µm)*	TOT MPs	Fragments	Fibers	MPs $Ind.^{-1}$	MPs mg^{-1}
05/12/2016	Como	0.0252	879	7248	200	22	18	4	0.025	0.873
07/12/2016	Lecco	0.0045	48	1050	450	18	8	10	0.373	4.000
15/02/2017	Lecco	0.5467	19071	7485	200	2	2	0	0.0001	0.004
15/02/2017	Lecco	0.0037	40	622	450	6	2	4	0.151	1.622
16/02/2017	Como	0.0086	300	2894	200	22	22	0	0.073	2.558
16/02/2017	Como	0.0049	52	252	450	0			0	<LOD
16/02/2017	Como	0.002	37	3	850	6	0	6	0.160	3.000
23/05/2017	Lecco	0.0304	1060	11223	200	12	12	0	0.011	0.395
23/05/2017	Lecco	0.0144	154	3213	450	26	22	4	0.169	1.806
23/05/2017	Lecco	0.0659	1236	1490	850	2	2	0	0.002	0.030
07/06/2017	Como	0.0274	956	19033	200	16	12	4	0.017	0.584
07/06/2017	Como	0.0084	90	4157	450	8	0	8	0.089	0.952
19/07/2017	Como	0.0486	1695	19033	200	18	18	0	0.011	0.370
19/07/2017	Como	0.0049	52	4157	450	4	0	4	0.076	0.816
19/07/2017	Como	0.016	300	45	850	2	0	2	0.007	0.125
29/08/2017	Lecco	0.0485	1692	7600	200	26	22	4	0.015	0.536
29/08/2017	Lecco	0.006	64	879	450	0			0	<LOD
15/11/2017	Lecco	0.0422	1472	6404	200	42	30	12	0.029	0.995
15/11/2017	Lecco	0.0057	61	233	450	22	14	8	0.360	3.860
16/11/2017	Como	0.0031	33	318	450	2	2	0	0.060	0.645
16/11/2017	Como	0.0011	21	17	850	4	4	0	0.194	3.636
07/02/2018	Como	0.0062	66	252	450	0			0	<LOD
07/02/2018	Como	0.0017	32	3	850	4	4	0	0.125	2.353
08/03/2018	Lecco	0.0159	555	16941	200	14	12	2	0.025	0.881
08/03/2018	Lecco	0.0058	62	2806	450	0			0	<LOD
26/04/2018	Lecco	0.0335	1169	16941	200	2	0	2	0.002	0.060
07/05/2018	Lecco	0.2367	8257	16941	200	44	34	10	0.005	0.186
07/05/2018	Lecco	0.0456	855	1259	850	8	0	8	0.009	0.175
05/06/2018	Como	0.0424	1479	18450	200	20	14	6	0.014	0.472
05/06/2018	Como	0.0146	156	1675	450	6	4	2	0.038	0.411
05/06/2018	Como	0.0587	1101	267	850	20	20	0	0.018	0.341
30/07/2018	Como	0.0658	2295	18450	200	16	14	2	0.007	0.243
30/07/2018	Como	0.0078	84	1675	450	4	2	2	0.048	0.513
06/08/2018	Lecco	0.0251	876	15605	200	18	14	4	0.021	0.717
06/08/2018	Lecco	0.0068	73	1545	450	2	0	2	0.027	0.294
28/08/2018	Como	0.0145	506	18450	200	4	0	4	0.008	0.276
26/11/2018	Lecco	0.0052	56	103	450	0			0	<LOD
26/02/2025	Como	0.0026	91	967	200	8	6	2	0.088	3.077
06/03/2025	Como	0.0122	426	709	200	34	22	12	0.080	2.787
02/04/2025	Como	0.0182	635	2942	200	24	20	4	0.038	1.319
23/06/2025	Como	0.0302	1053	2317	200	20	12	8	0.019	0.662
13/10/2025	Como	0.02465	860	2241	200	22	12	10	0.026	0.892

24/10/2025	Como	0.04744	1655	2893	200	40	40	0	0.024	0.843
04/11/2025	Como	0.004455	155	290	200	14	7.5	6.5	0.084	2.939
14/11/2025	Como	0.023	802	566	200	76	58	18	0.095	3.304
05/12/2025	Como	0.00817	285	3001	200	22	10	12	0.077	2.693
**Mean±SD**	**Lake Como**								**0.06±0.08**	**1.14±1.22**

*All different size net had a diameter of 52 cm.

Table S2. Information on zooplankton samples collected in 2025.

Date	Como Lake Branch	Hour	Mesh size (µm)	Coordinates	
26/02/2025	Como	12.00	200	45°48'59"N	9°4'3"E
06/03/2025	Como	14.45	200	45°49'2"N	9°4'5"E
02/04/2025	Como	14.30	200	45°49'4"N	9°4'5"E
23/06/2025	Como	14.45	200	45°49'3"N	9°4'6"E
13/10/2025	Como	15.00	200	45°48'56"N	9°4'49"E
24/10/2025	Como	14.45	200	45°49'19"N	9°4'6"E
04/11/2025	Como	15.00	200	45°48'55"N	9°4'49"E
14/11/2025	Como	15.15	200	45°48'55"N	9°4'38"E
05/12/2025	Como	10.20	200	45°48'56"N	9°4'49"E

Table S3. Seasonal variation in MP contamination of zooplankton (MPs ind.$^{-1}$ and MPs mg^{-1} dry weight) (years 2016, 2017, 2018 and 2025).

	Season			
	**Winter**	**Spring**	**Summer**	**Autumn**
**MPs Individual^{-1}**	0.098±0.109	0.034±0.053	0.026±0.025	0.097±0.114
**MPs mg^{-1}**	1.834±1.420	0.764±0.939	0.457±0.253	1.902±1.502

Table S4. Results of procedural blanks.

Blanks	Fragments	Fibers	Tot. MPs
Blank 1	0	0	0
Blank 2	1	0	1
Blank 3	1	0	1
Blank 4	5	1	6
Blank 5	3	0	3
Blank 6	2	1	3
**Mean±SD**	**2.00±1.79**	**0.33±0.52**	**2.33±2.22**
**LOD**			**8.99**
**LOQ**			**24.53**

Text S1. In this work, zooplankton observed exposure to MPs (MPs m^{-3}) was calculated as MPs ingested by zooplankton (MPs $individual^{-1}$) multiply per zooplankton abundance (individual m^{-3}) [1].

$$MPs\ m^{-3} = MPs\ individual^{-1}\ x\ individual\ m^{-3}$$

Text S2. Information on zooplankton density (individual m^{-3}) in Lake Como.

- Mean zooplankton density was 6125 ± 7938 ind. m^{-3} in the Como branch and 6241 ± 6466 ind. m^{-3} in the Lecco branch of Lake Como.
- Mean zooplankton density was 4687 ± 5985 ind. m^{-3} in 2017, 8210 ± 8425 ind. m^{-3} in 2018 and 1769 ± 1123 ind. m^{-3} in 2025.
- Mean zooplankton density was higher in summer (8334 ± 8102 ind. m^{-3}), followed by spring (7446 ± 7177 ind. m^{-3}), winter (2162 ± 2777 ind. m^{-3}) and autumn (1452 ± 2122 ind. m^{-3}).

References

1. Alfonso, M.B.; Lindsay, D.J.; Arias, A.H.; Nakano, H.; Jandang, S.; Isobe, A. Zooplankton as a Suitable Tool for Microplastic Research. *Science of The Total Environment* **2023**, *905*, 167329, doi:10.1016/j.scitotenv.2023.167329.
